# Neural substrates of purely endogenous, self-regulatory control of attention

**DOI:** 10.1038/s41598-018-19508-6

**Published:** 2018-01-17

**Authors:** Suk Won Han, Hyunji Shin, Dahee Jeong, Shinyoung Jung, Eunhee Bae, Joo Yeon Kim, Hyeon-Man Baek, Kyoheon Kim

**Affiliations:** 1 Department of Psychology, Chungnam National University, Daejeon, Republic of Korea; 2 Korea Basic Science Institute, Ochang, Chungbuk Republic of Korea; 3 Department of Molecular Medicine, Gachon University, Seongnam-si, Republic of Korea

## Abstract

Stimulus-driven orienting of attention toward a novel, salient stimulus is a highly adaptive behavior. In an opposing vein, it is also crucial to endogenously redirect attention to other stimuli of behavioral significance if the attended stimulus was evaluated to be unimportant. This stimulus-driven orienting and subsequent reorienting of attention are known to be mediated by similar neural substrates. However, this might be because reorienting was triggered by a sensory transition exogenously capturing attention, such as an abrupt onset of a new stimulus. Here, we used fMRI to measure the human brain’s activity when attention captured by a salient distractor is endogenously reoriented toward the concurrent main task, without any exogenous shifting of attention. As results, the transient activity of the anterior insula (AI) signaled such endogenous reorienting, predicting behavioral performance. This finding points to the central role of the AI in purely endogenous, self-regulatory control of attention.

## Introduction

A core function of the human information processing system is to configure a queue of discrete cognitive processes and behaviors to enable one to flexibly adapt to a dynamically changing environment. Specifically, in the face of a cognitively demanding task, a top-down task set is formulated, such that people focus attention on the task-related stimuli, becoming oblivious to other task-irrelevant sensory events. By this, the limited amount of processing resource is optimally allocated among multiple stimuli, protecting the system from information overload and distraction. However, the abrupt appearance of a novel, salient stimulus powerfully captures and takes attention away from the ongoing task. If the stimulus that captured attention is evaluated to be behaviorally significant, this stimulus should become a new target stimulus, captivating attention until this stimulus is acted upon. Otherwise, attention should be deviated from the stimulus and redirected to the original task.

While the capture of attention by salient stimuli takes place in a bottom-up or exogenous manner^1–4^, the specific nature of the process of reorienting attention from the salient distractor to the main task remains unclear. Extant studies have suggested that the reorienting process is mediated by similar neural substrates underlying stimulus-driven orienting of attention^3–6^. However, this might be because reorienting was initiated by a sensory event triggering the exogenous capture of attention. Specifically, in a predominantly used paradigm to clarify the neural substrates of reorienting, a salient, but task-irrelevant cue stimulus abruptly appears in periphery, followed by the presentation of a target stimulus. If the cue and target locations do not match, attention initially captured by the cue should be reoriented to the target location. In this case, reorienting is not clearly differentiated from the initial orienting; both processes are initiated by a salient sensory event, the abrupt appearance of stimuli (cue or target), exogenously capturing attention.

**Figure 1:**
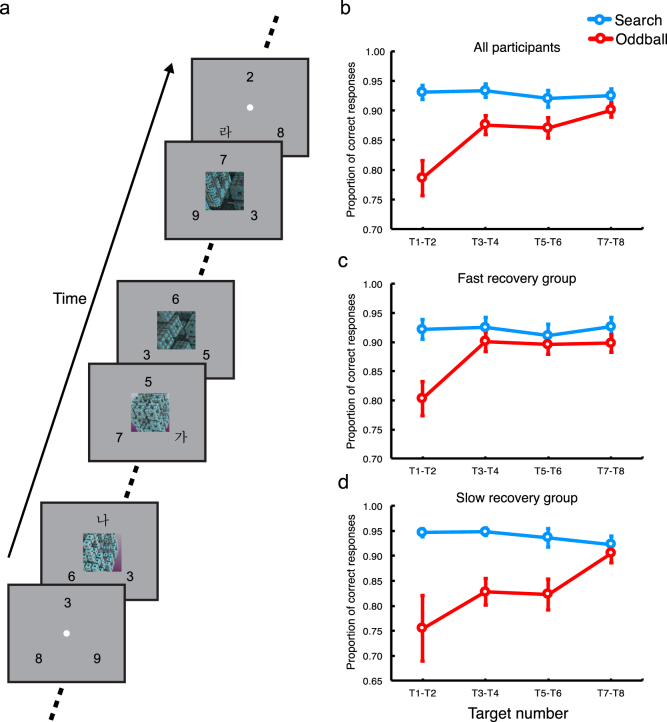
Example of trials and behavioral results. (**a**) An example of oddball trials. (**b**) Target accuracy data of all participants. (**b**) Target accuracy data of the fast recovery group. (**c**) Target accuracy data of the slow recovery group. Error bars represent standard errors of the mean.

The present study aimed at elucidating neural substrates of endogenous control of attention without any exogenous shifting of attention triggered by sensory transitions. To do so, in the experiment, participants were required to detect and identify multiple targets imbedded in three rapid serial visual presentations of distractors in periphery (Fig. 1a). A trial lasted 24 seconds, challenging the ability to sustain attention on the main task. While participants were engaged in the attention-demanding task, occasionally and unexpectedly, a temporally extended oddball stimulus (16-sec long movies) was presented at the center, simultaneously with the RSVP. The onset of the salient oddball movie should capture attention. However, participants would have to reorient attention from the task-irrelevant movie distractor to the main task, while the movie is still being played. In this case, reorienting is not triggered by any change in sensory stimulation, but should be solely evoked by participants’ endogenous control of attention. Given that this reorienting signal should be transient by nature^4,7,8^, brain regions involved in reorienting should elicit transient activity at some point in the midst of the oddball presentation.

Based upon a previous work employing the temporally extended oddball presentation^4^, we focused on activation profiles of the anterior insula (AI), anterior cingulate cortex (ACC), and inferior frontal junction (IFJ), as these were found to show transient activity when attention should be switched between the onset/offset of the oddball and task stimuli. We also examined how the temporoparietal junction (TPJ), which has been traditionally viewed as a region for reorienting, is activated when the reorienting process is required.

## Results

In each trial lasting 24 seconds, a total of 8 targets were presented with 2–4 seconds of inter-target intervals. To assess participants’ task performance for the search and oddball trials, we calculated proportions of correct responses for each target (T1~T8). To increase statistical power, target accuracies for each consecutive pair of targets (T1-T2, T3-T4, T5-T6, and T7-T8) were averaged. Then, we compared target accuracy between the search and oddball trials.

For the search trials, participants’ target accuracy was high throughout the trial duration (Fig. 1b). By contrast, target performance for the oddball trials was severely impaired at the early phase of the trial, but gradually recovered. To statistically assess this pattern, a repeated measures two-way ANOVA was applied to target accuracy data with target pair (1st, 2nd, 3rd, and 4th) and trial type (search and oddball) as factors. This analysis revealed significant main effects of both factors, p’s < 0.00005. The interaction between the factors was also significant, F(3, 57) = 12.32, p < 0.00005. Specifically, accuracy for the pair of T1 and T2 (T1-T2) for the oddball trials was significantly lower than that for the search trials, t(19) = 5.48, p < 0.0001. The differences between the search and oddball trials for the pairs of T3-T4 and T5-T6 were also significant, p’s < 0.01, with significantly greater difference for the T3-T4 pair than that for T5-T6, p’s < 0.00003. Target accuracy for the pair of T7-T8 did not differ across the trial types, p > 0.09. These findings demonstrate that at the early phase of the oddball presentation, attention was captured by the oddball, interfering with the target search. However, participants endogenously reoriented their attention from the oddball to the main task while the oddball was still being presented.

Importantly, there was much individual difference regarding the time point at which target performance impaired by the oddball presentation recovers (Fig. 1c and d). To examine this, we estimated the inflection point from which the magnitude of distractor interference does not further decrease for each individual participant. Specifically, for each individual participant, we calculated the magnitude of distractor interference by subtracting oddball trial accuracy from search trial accuracy for each target pair, which produced a 1 × 4 vector (one-dimensional array). This data was smoothed using the loess function implemented in R package. Then, we defined the inflection point as a point where the sign of distractor interference value changed from positive to negative.

**Figure 2:**
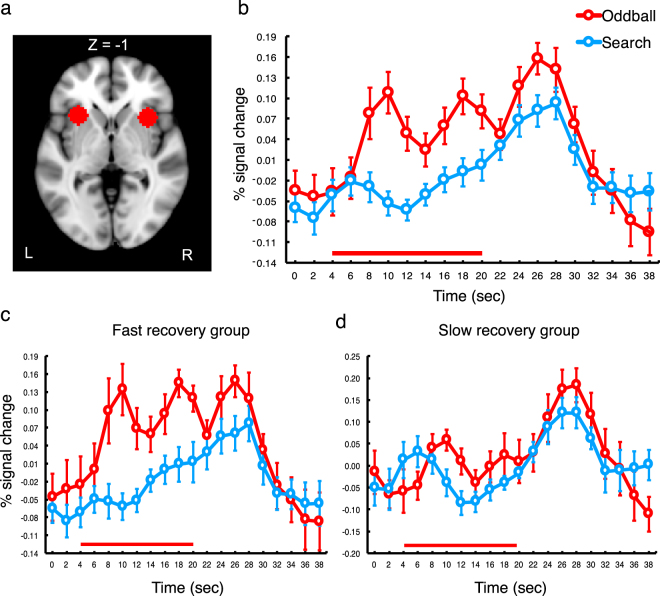
Anatomical location and activation timecourse of the AI. (**a**) Anatomical location of the AI. MNI coordinates (x, y, z): right/left, 34/−30, 16/18, −2/0. (**b**) Activation timecourse of the AI for the oddball and search trials. (**c**) AI activation timecourse of the Fast recovery group. (**d**) AI activation timecourse of the Fast recovery group. Error bars represent standard errors of the mean. The red bars on the plots represent the presentation of the 16-sec long oddball distractor.

This estimation procedure revealed that a group of participants (N = 7) showed particularly slow recovery (Fig. 2d); their target performance did not fully recover until the fourth pair of targets (T7-T8) were presented. By contrast, the remaining participants’ performance relatively quickly recovered by the time at which the second pair (N = 7) or third pair (N = 5) of targets were presented. There was also a participant whose performance was not impaired by the oddball at all. To quantitatively assess this individual variability, we classified the former group into the slow recovery group, while the rest was assigned into the fast recovery group. When a three-way mixed ANOVA with target pair (1st, 2nd, 3rd, and 4th), trial type (search and oddball) as within subject factors and recovery speed (fast and slow) as a between subject factor was applied to accuracy data, the main effects of trial type and target pair were significant, p’s < 0.00005. The interactions between trial type and recovery speed and between target pair and trial type were also significant, p’s < 0.05. Finally, the three way interaction between the factors was significant, F(3, 54) = 2.99, p < 0.05.

To sum up, the presentation of oddball during the target search severely impaired target processing at the early phase of the trial. However, search performance gradually recovered before the oddball presentation was finished, suggesting that participants endogenously reoriented their attention from the oddball to the main task. We also found significant inter-individual variability in how quickly this endogenous reorienting took place.

To investigate neural substrates of endogenous reorienting of attention from the oddball to the main task, we probed brain regions found to be activated greater for the oddball trials than for the search trials. Among the significant activational foci yielded by the contrast between the oddball and search trial activities, our analyses were focused on activation profiles of anterior insula (AI), anterior cingulate cortex (ACC), inferior frontal junction (IFJ), and temporoparietal junction (TPJ). There are two reasons for why these regions were prioritized for the ROI analyses. First, these are well known to be involved in oddball processing^2–4,9,10^. Especially, the TPJ has been suggested to be a region associated with the process of reorienting of attention from attention-capturing sensory events to the target stimulus. Second, our previous work employing the temporally extended oddball showed that the AI, ACC, and IFJ were transiently activated at the onset and offset of the oddball movie, implicating these in orienting attention toward the oddball and reorienting from the oddball to the main task^4^.

As shown in Fig. 2a and b, the AI showed transient responses to the onset of the oddball movie, implicating this region in the capture of focal attention by a salient sensory event^4,11,12^; the signal for the oddball trial initially peaked 10 seconds after the trial onset (6 seconds after the oddball onset), and then, quickly dropped to baseline 14 seconds after the trial onset. Remarkably, the AI activity rose again from this time point and a second peak was observed 18 seconds after the trial onset, followed by another quick drop. There was a third peak 28 seconds after the trial onset (24 seconds after the oddball onset), evoked by the trial offset and oddball offset.

This triple-peaked pattern of the oddball response was statistically assessed via t-tests. As results, the initial peak amplitude was significantly greater than the signal amplitude at the first inter-peak volume (between the first and second peak), t(19) = 3.13, p < 0.01. The second peak amplitude was also significantly greater than those at the first inter-peak and second inter-peak volumes (between the second and third peak), p’s < 0.005. A third peak response observed 26 seconds after the trial onset (6 seconds after the oddball offset) was also significantly greater than the second inter-peak activity, t(19) = 6.69, p < 0.000005.

The first peak of the AI activity should be associated with the capture of focal attention by the onset of the salient oddball movie, consistent with many previous studies^4,5,11,12^. The third peak signals the end of the oddball movie 4. While these peaks were elicited by the sensory transitions at the oddball onset and offset, respectively, there was no sensory transition associated with the second peak. We interpret that this second peak is associated with endogenous reorienting of attention from the oddball distractor to the main task. In the absence of sensory transition, to perform the task, participants would have to intentionally reorient attention from the salient distractor or change mode from oddball processing to target search. We suggest that the transient activity in the midst of oddball presentation signals such endogenous control of attention. Supporting this claim, the AI activity of participants whose search performance quickly recovered (fast recovery group, see above) showed a robust triple-peaked response (significant second peak), p’s < 0.005 (Fig. 2c). By contrast, the AI activity of participants whose search performance did not fully recover until the last pair of targets was presented showed no significant activity in the middle of the oddball presentation (Fig. 2d).

**Figure 3:**
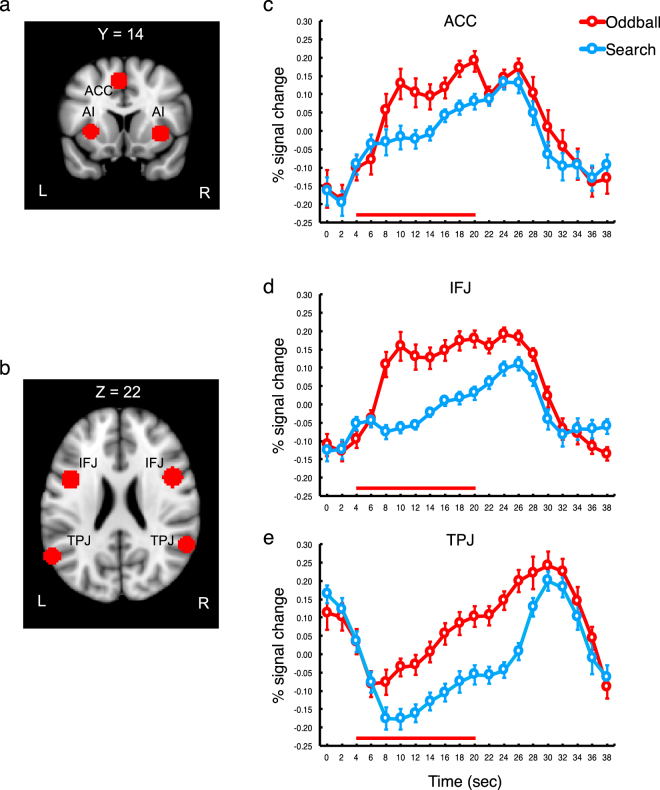
Anatomical locations and timecourse activation of the ACC, IFJ and TPJ. (**a**) Anatomical location of ACC. MNI coordinates (x, y, z): −4, 10, 48. (**b**) Anatomical locations of the IFJ and TPJ. MNI coordinates of the IFJ (x, y, z): right/left, 48/−42, 8/6, 23/26. MNI coordinates of the TPJ (x, y, z): right/left, 58/−58, −50/−60, 18/18. (**c**) Activation timecourse of the ACC. (**d**) Activation timecourse of the IFJ. (**e**) Activation timecourse of the TPJ. Error bars represent standard errors of the mean. The red bars on the plots represent the presentation of the 16-sec long oddball distractor.

Other regions than the AI showed primarily sustained responses to the oddball, with no difference between the groups. While the ACC (Fig. 3a) activity showed a pattern of triple-peaked response, this triple-peaked pattern was not significant. Specifically, the difference between the first peak amplitude and the first inter-peak amplitude was not significant, p > 0.32, nor was the difference between the second peak amplitude and the second inter-peak amplitude, p > 0.08 (Fig. 3c).

The activity of the IFJ was also sustained for the oddball and search trials, with significantly greater activity for the oddball trials (Fig. 3d). Specifically, the amplitudes of the oddball activity were significantly greater than those of the search activity from 8 seconds after the trial onset (4 seconds after the oddball onset) to 24 seconds after the trial onset (20 seconds after the oddball onset), p’s < 0.000065. This is a consistent result with our previous study^4^. The sustained IFJ activity evoked by the oddball presentation might reflect the evaluative process of the salient oddball stimuli or the process of protecting the task representation from distractor interference^4,13–15^. Specifically, in the oddball trials of the present experiment, participants had to search the RSVP while the oddball movie was being played. In this case, the process of suppressing the distractor should be sustained throughout the oddball presentation.

The TPJ activity was also significantly greater for the oddball trials than for the search trials, p’s < 0.005 (Fig. 3e). In particular, the performance of the top-down search task deactivated this region; the signal amplitudes were significantly below zero from 8 seconds after the trial onset to 16 seconds after the trial onset, p’s < 0.0005, which is consistent with many previous studies^4,16–18^. This deactivation was significantly attenuated when the oddball was presented. This attenuation of TPJ deactivation might be the product of combination of positive activation of the TPJ by the oddball presentation and negative activation (suppression) imposed on this region by the top-down search process. Specifically, we previously showed that the presentation of temporally extended oddball yielded sustained activation of the TPJ^4^. Notably, in that study, the oddball presentation and the task stimuli presentation did not temporally overlap; the oddball presentation interrupted the presentation of search stimuli. Hence, during the oddball presentation, the search might not have been performed. By contrast, in the present study, the oddball and the task stimuli were simultaneously presented. In this case, the positive activation evoked by the oddball might have been cancelled out by the suppression of the TPJ elicited by the performance of the concurrent search task.

Finally, we also probed the FEF, IPS, and extrastriate visual cortex, which were found to be activated greater for the oddball trials than the search trials. All these regions showed single-peaked, sustained responses to the oddball presentation, similarly with the IFJ and ACC. The sustained activation of the FEF and IPS might be because goal-directed search process still proceeded during the oddball presentation^4^, while the extrastriate cortex activity was due to the high saliency of the oddball movies.

**Figure 4:**
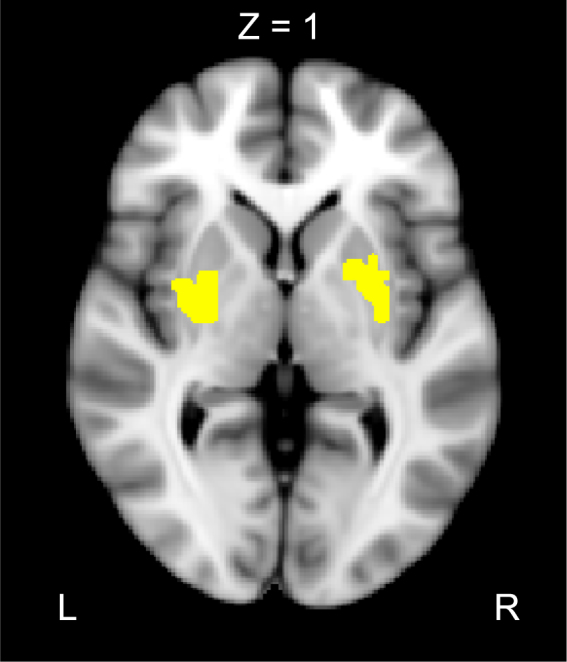
Result of the PPI analysis using the AI as a seed region. Significant clusters were found on bilateral putamen. MNI coordinates (x, y, z): right/left, 28/−28, −1/2, −3/−2.

Having shown that the AI is a core node of endogenous reorienting of attention, we ran a psycho-physiological interaction (PPI) analysis to identify brain regions, whose connectivity with the AI differs, depending on whether only the task stimuli are presented (search trial) or temporally extended oddball distractors are simultaneously presented with the task stimuli (oddball trial). This PPI analysis yielded significant activation foci on bilateral putamen, indicating that the functional connection between the AI and the putamen was significantly greater for the oddball trials than for the search trials (Fig. 4). This result is consistent with a growing number of findings that the context-dependent (trial type-dependent) corticostriatal connectivity is crucial for attentional control^19–23^.

## Discussion

The present study aimed at clarifying the neural substrates of endogenous orienting of attention in the absence of sensory transition. We found that while participants try to focus on the main task, overcoming interference exerted by attention-capturing distractors, a transient activity was evoked from the AI in the middle of sustained presentation of the oddball distractor. Specifically, we found that the AI was transiently activated by the onset and offset of the salient, movie distractors, in line with a previous study^4^. What is novel in the present study is that the long-lasting movie distractors were simultaneously presented with the task stimuli. In this case, to successfully accomplish the task, participants had to instantly reorient their attention from the distractor to the main task. We suggest that the transient AI activity in the midst of oddball presentation signals this reorienting process. Supporting this, the transient AI activity in the middle of the sustained presentation of distractors was observed only from the brain responses of participants who quickly reoriented attention from the distractor to the main task. By contrast, when the reorienting did not take place until the distractor presentation was almost finished, there was significant AI activity only at the onset and offset of the distractor, with no significant activity while the distractor movie was being played. It is important to note that the observed AI activity in the midst of the distractor presentation was not evoked by any drastic sensory transition. This demonstrates that the AI can be transiently activated solely by endogenous, self-regulatory control of human observers.

The present finding fits well with recent studies showing that the AI plays a crucial role in switching between two distinct brain networks^11,24^. Specifically, Sridharan and colleagues showed that the AI was critically involved in orchestrating two distinct functional networks of the human brain, the central-executive network (CEN) and the default-mode network (DMN). According to this study, when people are in rest, the default mode network is activated. However, when a salient stimulus appears, the activation of the default mode network should cease, and the central executive network should be activated to handle the stimulus. Importantly, a third network, the salience network, having the AI as a core node, plays a role in switching between the CEN and the DMN. In line with this switching role of the AI, the present study showed that when participants who were engaged in oddball processing decided to reorient attention to the main task, the AI was transiently activated. This finding provides additional evidence for the claim that the AI plays a role in switching between different modes (oddball processing vs. target processing or default mode vs. attention mode).

In addition to the switching process, the AI has been associated with the capture of focal attention, the initiation of task control, and the maintenance of alertness for behaviorally significant events^5,11,12,25–27^. Besides these, the present study adds that the AI is involved in the initiation of attentional control in a purely endogenous manner without any external sensory events triggering attentional capture. Considering all these findings, we suggest that the AI is a key hub region in the human brain to initiate a queue of cognitive processes required for adaptive behaviors. Specifically, when a salient sensory event occurs, this should be detected and the information processing system should be alerted to timely handle the event. We suggest that an important role of the AI is to alert the system of the salient sensory event, initiating a series of further processes. Once the AI alerts the system, attention should be rapidly oriented toward the sensory event. This account can explain a large body of previous studies, which associate the AI with the transient capture of focal attention, maintaining vigilance, and many socio-emotional processing^5,11,12,25–30^. Expanding these findings, we provide novel evidence that the AI is also involved when humans initiate cognitive control in a purely endogenous, self-regulatory manner even without any salient sensory event.

Other prefrontal regions, the IFJ and ACC, showed sustained responses while the task stimuli and distractors were simultaneously presented, with significantly greater activity for the oddball distractors. These findings are also consistent with a large body of previous studies suggesting that these regions are particularly sensitive to the presentation of salient stimuli^2–4,9,16^. Related to this, the elevated and sustained activity by the oddball distractor might reflect these regions’ role in cognitive control to maintain the task set, overcoming distractor interference^13,14^.

The activation profile of the TPJ is also remarkable. The simultaneous presentation of the oddball distractor and task stimuli in a temporally extended manner elevated the activation level of the TPJ, but the TPJ activity did not increase above the baseline. This is a contrasting result from a previous study, which also employed the temporally extended oddballs^4^. In that study, throughout the 10-sec duration of the oddball movie presentation, robust sustained activation above baseline was found in the TPJ. This sustained TPJ activation was associated with the process of stimulus evaluation, consisting of identifying and categorizing the oddball stimuli. Importantly, this sustained TPJ activity was found when the presentation of the task stimuli was interrupted for 10 seconds by the oddball presentation. By contrast, in the present study, the search stimuli and oddballs were simultaneously presented. Under this experimental setting, the TPJ activation level did not increase beyond baseline. Presumably, this is because the ongoing search activity imposed suppression of the TPJ activity, cancelling out positive activation evoked by the oddball presentation.

It is also important to note that the functional connection between the AI and the striatum increased when the temporally extended oddball accompanied the task stimuli, taxing the processes of overcoming distractor interference and reorienting of attention. Recently, a growing number of studies implicate the striatum in various types of cognitive/attentional control. Specifically, the striatal activity has been found to be associated with attentional shifting, suppressing task-irrelevant information, and selective gating of information in working memory^19–23^. The present functional connectivity result also supports the contribution of the striatum to cognitive control. An important point here is that the present study found a functional coupling between the AI and the striatum to meet the increased demand for endogenous control of attention, while previous studies have focused on the striatum’s functional connectivity with the lateral prefrontal cortex.

Finally, the current experimental paradigm has much potential for clinical application. Recently, much work has been devoted to clarify the association between attentional mechanisms and clinical disorders, such as substance addiction, autism, and attention deficit hyperactivity disorder. A working hypothesis is that members of those clinical populations might show impairment in the processes of attentional allocation, shifting, or reorienting. To test this, conventional attentional paradigms, in which cue and target stimuli are transiently presented, were used^31,32^. We believe that these kinds of paradigms evoke exogenous capture of attention, obscuring the deficit in endogenous control of attention. Here we suggest that a paradigm straining purely endogenous and self-regulatory control of attention should be developed for effective diagnosis and treatment of attention-related clinical disorders and the present paradigm can be one of such paradigms.

Taken together, the present study clarified neural substrates of purely endogenous, self-regulatory control of attention. Even though the endogenous reorienting of attention from salient distractors to the main task is a fundamental process of human cognition, limitations of the extant paradigms impeded distinguishing the reorienting activity from the activity for stimulus-driven orienting of attention. Here, using an innovative experimental paradigm, we found that the AI was transiently activated when the process of reorienting took place. This reorienting was not triggered by any sensory transition, but by participants’ intention. This finding significantly expands the present understanding of the AI function. Extant studies have associated the AI with encoding stimulus saliency, bottom-up capture of focal attention, switching between differential functional brain networks, and various socio-affective processing. Here, we provide novel evidence that the AI contributes to the initiation of endogenous control of attention. Given that this endogenous control is the foremost and fundamental process in human cognition, the present finding places the AI on the role of a functional hub initiating cognitive control for adaptive behaviors.

## Methods

### Participants

Twenty adults (six males, aged 23–33) participated in the study for monetary compensation. This sample size was based upon a previous study employing a similar experimental paradigm^4^. Specifically, N of 14 was sufficient to detect a transient signal while distractor movie is being played. Indeed, a power analysis using this dataset revealed that N of 6 was sufficient to detect significant effect at the level of 0.80. To further ensure that the present study is powerful enough, we decided to collect 20 participants’ data. The Chungnam National University Institutional Review Board approved the experimental protocol and written informed consent was obtained from each participant. The study was performed in accordance with the approved guidelines.

### Behavioral paradigm

The experiment was designed and run using Psychopy^33^. The task consisted of detecting and identifying Korean letters embedded in three rapid visual presentation (RSVP) of digits. Each RSVP was presented on three locations evenly spaced on an imaginary circle, whose radius was 5 degree of visual angle. Each frame in the RSVPs lasted 200 ms. The target letters (‘가’, ‘나, ‘다’, or ‘라’) could be presented in any of the three RSVPs. A single trial, which included 8 targets, lasted 24 seconds. Each target stimulus was separated by a 2 or 4-sec interval, during which distractors were presented.

Importantly, in 24 trials out of a total of 96 trials, an oddball movie lasting 16 seconds was presented at the center of the screen 4 seconds after the trial onset, simultaneously with the RSVPs (Oddball trials). The set of oddball stimuli comprised movies depicting non-meaningful, abstract animations (e.g. continuously transforming fractals, molecular polymerization, swirling waves, constantly rotating color patches in random direction, evolving line drawings of geometric shapes, dynamically transforming objects, moving flashlight in random direction, or continuously evolving colored geometric shapes) and movies depicting real-world situations (fast-moving roller coasters, overturning ships, moving toys, a remote-controlled vacuum cleaner, a dogfight of jet-fighters, online gaming, and a laptop commercial). The abstract oddballs and real-world oddballs did not yield differential results^4^. The remaining 72 trials did not include any oddball (Search trials).

### fMRI methods

All the imaging parameters and preprocessing steps were identical to those of our previous work^4^. Specifically, anatomical 2D and 3D high-resolution T1-weighted images were acquired with conventional parameters on a 3 T Philips scanner at Korea Basic Science Institute. For the functional scan, thirty-three 3.5 mm axial slices (0.5 mm skip; 3.75 × 3.75 mm in-plane) were taken parallel to the AC-PC line (TR, 2000 ms; TE, 35 ms; FA, 79°; FOV, 240 mm), for a total of 255 brain volumes per fMRI run. There were 6 functional runs, each of which included 16 trials. A blank interval of variable duration following an exponential distribution (9 trials × 4 sec, 5 trials × 8 sec, 2 trials × 12 sec) was inserted between each trial.

Imaging data were analyzed using FSL (http://fsl.fmrib.ox.ac.uk). Data preprocessing included non-brain removal to improve image registration, slice scan time correction, 3D motion correction, high-pass filtering (100-sec period cutoff), and spatial smoothing with a 5-mm Gaussian kernel (FWHM). All functional data of each participant were co-registered to each individual’s anatomical T1-weighted image, and transformed into Montreal Neurological Institute standard brain.

To isolate regions of interest (ROIs), a conventional generalized linear model analysis was performed. Specifically, regressors were defined for each trial type (search and oddball trials) and convolved with a double-gamma function. Then, we ran a group random effect analysis, contrasting search trial activity with oddball trial activity. The resulting statistical parametric map (SPM) was thresholded using clusters determined by a voxelwise Z threshold of 2.3 and a cluster significance threshold of P = 0.05, corrected for whole-brain multiple comparisons. This SPM analysis yielded significant activational foci on the core nodes of the stimulus-driven attention network and salience network, comprising inferior frontal junction (IFJ), temporoparietal junction (TPJ), anterior insula (AI), and anterior cingulate cortex (ACC). Regions in the dorsal, frontoparietal attention network, frontal eye fields (FEF) and intraparietal sulcus (IPS), were also activated. We also found extensive activation clusters on extrastriate visual cortex. Among these activational foci, subsequent ROI analyses were focused on the regions included in the stimulus-driven attentional network or salience network, such as AI, ACC, IFJ, and TPJ^2,3,10^.

For ROI analyses, event-related time course of blood oxygenated level dependent (BOLD) signal for each participant and trial type was calculated and plotted as percent signal change relative to the mean of the run. To examine the full temporal profile of activities of the ROIs, we used data from 19 volumes from the trial onset. Given that the TR of the present experiment was 2 seconds, we plotted the timecourse data from the trial onset up to 38 second after the onset. This was to ensure to capture the full temporal profile of activities evoked by the long-lasting trial of the present experiment. The activation timecourses were averaged across participants, yielding group-averaged timecourses. As no hemispheric difference was found, timecourses of bilateral ROIs were collapsed to increase power.

Having found that the AI was associated with the endogenous reorienting of attention from the distractor to the main task (see Results), we ran a generalized psychophysiological interaction (gPPI) analysis using this region as a seed region^34^. Specifically, a generalized linear model was constructed with a physiological regressor, time series data extracted from the AI, two task regressors, each of which is associated with the search and oddball trials, and two PPI regressors. The PPI regressors were the interaction terms between the task regressors and the physiological regressor, computed as the product of the physiological regressor and the task regressors. Then, we ran a contrast between the PPI regressors to identify regions, whose connectivity with the AI differs across trial types (Oddball vs. Search trials), while regressing out activities associated with task regressors. The resulting SPM was thresholded using clusters determined by a voxelwise Z threshold of 2.3 and a cluster significance threshold of P = 0.05, corrected for whole-brain multiple comparisons. This analysis was done separately for the left and right AIs. The result did not differ, depending on whether the seed was the right or left AI. We also ran another PPI analysis, in which the PPI regressor was computed as the product of the AI activity and a difference vector of the oddball and search trials (Oddball - Search). The results remained the same.

### Data availability

All the data and analysis scripts are available upon reasonable requests.

## References

[1] Chica AB, Bartolomeo P, Valero-Cabre A (2011). Dorsal and ventral parietal contributions to spatial orienting in the human brain. J Neurosci.

[2] Corbetta M, Patel G, Shulman GL (2008). The reorienting system of the human brain: From environment to theory of mind. Neuron.

[3] Corbetta M, Shulman GL (2002). Control of goal-directed and stimulus-driven attention in the brain. Nature Reviews Neuroscience.

[4] Han SW, Marois R (2014). Functional fractionation of the stimulus-driven attention network. J Neurosci.

[5] Trautwein FM, Singer T, Kanske P (2016). Stimulus-Driven Reorienting Impairs Executive Control of Attention: Evidence for a Common Bottleneck in Anterior Insula. Cereb Cortex.

[6] Kincade JM, Abrams RA, Astafiev SV, Shulman GL, Corbetta M (2005). An event-related functional magnetic resonance imaging study of voluntary and stimulus-driven orienting of attention. J Neurosci.

[7] Konishi S (1998). Transient activation of inferior prefrontal cortex during cognitive set shifting. Nature Neuroscience.

[8] Yantis S (2002). Transient neural activity in human parietal cortex during spatial attention shifts. Nature Neuroscience.

[9] Downar J, Crawley AP, Mikulis DJ, Davis KD (2000). A multimodal cortical network for the detection of changes in the sensory environment. Nature Neuroscience.

[10] Seeley WW (2007). Dissociable intrinsic connectivity networks for salience processing and executive control. J Neurosci.

[11] Menon V, Uddin LQ (2010). Saliency, switching, attention and control: a network model of insula function. Brain Struct Funct.

[12] Nelson SM (2010). Role of the anterior insula in task-level control and focal attention. Brain Struct Funct.

[13] Brass M, Derrfuss J, Forstmann B, von Cramon DY (2005). The role of the inferior frontal junction area in cognitive control. Trends in Cognitive Sciences.

[14] Derrfuss J, Brass M, Neumann J, von Cramon DY (2005). Involvement of the inferior frontal junction in cognitive control: Meta-analyses of switching and Stroop studies. Human Brain Mapping.

[15] Muhle-Karbe PS (2015). Co-Activation-Based Parcellation of the Lateral Prefrontal Cortex Delineates the Inferior Frontal Junction Area. Cereb Cortex.

[16] Asplund CL, Todd JJ, Snyder AP, Marois R (2010). A central role for the lateral prefrontal cortex in goal-directed and stimulus-driven attention. Nature Neuroscience.

[17] Shulman GL, Astafiev SV, McAvoy MP, d’Avossa G, Corbetta M (2007). Right TPJ deactivation during visual search: Functional significance and support for a filter hypothesis. Cerebral Cortex.

[18] Todd JJ, Fougnie D, Marois R (2005). Visual short-term memory load suppresses temporo-parietal junction activity and induces inattentional blindness. Psychological Science.

[19] Chatham CH, Frank MJ, Badre D (2014). Corticostriatal output gating during selection from working memory. Neuron.

[20] van Schouwenburg MR, O’Shea J, Mars RB, Rushworth MF, Cools R (2012). Controlling human striatal cognitive function via the frontal cortex. J Neurosci.

[21] van Schouwenburg MR, den Ouden HE, Cools R (2010). The human basal ganglia modulate frontal-posterior connectivity during attention shifting. J Neurosci.

[22] Westbrook A, Braver TS (2016). Dopamine Does Double Duty in Motivating Cognitive Effort. Neuron.

[23] Robertson BD, Hiebert NM, Seergobin KN, Owen AM, MacDonald PA (2015). Dorsal striatum mediates cognitive control, not cognitive effort per se, in decision-making: An event-related fMRI study. Neuroimage.

[24] Sridharan D, Levitin DJ, Menon V (2008). A critical role for the right fronto-insular cortex in switching between central-executive and default-mode networks. Proceedings of the National Academy Sciences of the United States of America.

[25] Dosenbach NUF, Visscher KM, Palmer ED, Miezin FM (2006). A core system for the implementation of task sets. Neuron.

[26] Dubis JW, Siegel JS, Neta M, Visscher KM, Petersen SE (2014). Tasks Driven by Perceptual Information Do Not Recruit Sustained BOLD Activity in Cingulo-Opercular Regions. Cereb Cortex.

[27] Sadaghiani S, D’Esposito M (2015). Functional Characterization of the Cingulo-Opercular Network in the Maintenance of Tonic Alertness. Cereb Cortex.

[28] Adolphs R (2002). Neural systems for recognizing emotion. Current opinion in neurobiology.

[29] Singer T, Critchley HD, Preuschoff K (2009). A common role of insula in feelings, empathy and uncertainty. Trends in Cognitive Sciences.

[30] Singer T, Lamm C (2009). The social neuroscience of empathy. Ann N Y Acad Sci.

[31] Grubb MA (2013). Endogenous spatial attention: evidence for intact functioning in adults with autism. Autism Res.

[32] Fischer J (2016). Unimpaired attentional disengagement in toddlers with autism spectrum disorder. Dev Sci.

[33] Peirce JW (2007). PsychoPy–Psychophysics software in Python. Journal of neuroscience methods.

[34] McLaren DG, Ries ML, Xu G, Johnson SC (2012). A generalized form of context-dependent psychophysiological interactions (gPPI): A comparison to standard approaches. NeuroImage.

